# ERAC knowledge, attitudes, and practices among obstetrics and gynecology medical staff

**DOI:** 10.3389/fpubh.2026.1786598

**Published:** 2026-04-10

**Authors:** Jiawei Chen, Yannan Li, Lu Wang, Weirong Gu, Rong Hu, Hao Zhu

**Affiliations:** 1 Department of Anesthesiology, Obstetrics & Gynecology Hospital of Fudan University, Shanghai Key Lab of Reproduction and Development, Shanghai Key Lab of Female Reproductive Endocrine Related Diseases, Shanghai, China; 2 Department of Obstetrics, Obstetrics & Gynecology Hospital of Fudan University, Shanghai Key Lab of Reproduction and Development, Shanghai Key Lab of Female Reproductive Endocrine Related Diseases, Shanghai, China

**Keywords:** cesarean section, enhanced recovery after surgery, knowledge, attitude, practice, physician, survey and questionnaire

## Abstract

**Introduction:**

This study aimed to assess the knowledge, attitudes, and practices (KAP) of obstetrics and gynecology medical staff concerning enhanced recovery after cesarean section (ERAC).

**Methods:**

A multi-center cross-sectional survey was conducted between February 22 and March 31, 2025, in 10 hospitals located across various regions of China. A structured questionnaire was employed to collect data on participants’ demographic characteristics and assess their scores in the domains of knowledge, attitude, and practice related to ERAC. The cutoff score for each domain was set at 80% of the total possible score to categorize levels as “good” or “poor.”

**Results:**

A total of 766 valid responses were obtained, yielding a valid questionnaire rate of 85.97%. Among the respondents, 685 (89.43%) were female, and 276 individuals (36.03%) had previously received ERAC-specific training. The average scores were 14.64 ± 5.93 (range: 0–25) for knowledge, 44.18 ± 5.45 (range: 10–50) for attitude, and 32.33 ± 10.30 (range: 10–50) for practice. Structural equation modeling suggested that knowledge was significantly associated with attitude (*β* = 0.441, *p* = 0.009) and practice (*β* = 0.501, *p* = 0.005), while attitude was also associated with practice (*β* = 0.203, *p* = 0.008). In addition, knowledge showed an indirect association with practice through attitude (*β* = 0.089, *p* = 0.006).

**Discussion:**

Despite generally positive attitudes, obstetrics and gynecology medical staff demonstrated insufficient knowledge and suboptimal practices concerning ERAC. Targeted educational interventions and systematic ERAC training programs are urgently needed to bridge the knowledge-practice gap and promote consistent implementation of evidence-based recovery protocols across diverse clinical settings.

## Introduction

Cesarean section (CS) rates have escalated globally, with current averages around 21% and projections to reach 30% by 2030, representing approximately 38 million procedures annually worldwide (1, 2). While CS rates vary significantly between countries, ranging from merely 5% in South Sudan to 58.9% in the Dominican Republic, China’s rates substantially exceed the World Health Organization’s recommendations, with more than 28% of procedures being performed (3, 4). This persistent increase has not only imposed significant burdens on healthcare systems but also exposes mothers to higher risks of surgical complications (15–20% higher than vaginal delivery), prolonged postoperative healing time (average recovery period of 4–6 weeks versus 1–2 weeks for vaginal birth), and increased rates of postpartum infections (5–8% versus 1–3%), posing substantial physical and financial challenges for both patients and healthcare facilities (5, 6).

Enhanced Recovery After Cesarean (ERAC) protocols represent a multimodal and multidisciplinary approach to optimizing perioperative management, with evidence demonstrating reduced hospital stays (by approximately 13.78 h), decreased opioid consumption, and maintained safety without increased readmission rates or complications (7). These protocols encompass standardized interventions across pre-, intra-, and postoperative phases, aiming to reduce surgical stress response, accelerate functional recovery, and improve maternal outcomes (5). Professional societies have delineated comprehensive recommendations, with the Society for Obstetric Anesthesia and Perinatology (SOAP) identifying 25 specific elements across all perioperative phases (8).

Despite established guidelines and demonstrated benefits, ERAC implementation varies considerably across healthcare settings, particularly in China where adoption remains suboptimal (9). The Knowledge, Attitude, and Practice (KAP) model provides a framework for understanding implementation barriers, suggesting that healthcare behaviors are fundamentally determined by providers’ knowledge, perceptions, and attitude (10, 11). Obstetrics and gynecology medical staff represent the primary executors of ERAC protocols, yet previous investigations have revealed that many components remain unimplemented, with potential barriers including varied awareness levels, attitudinal differences, resource constraints, and challenges in interdisciplinary collaboration (12). While substantial research has documented patient outcomes (13), there exists a notable gap in understanding Chinese healthcare providers’ knowledge, attitudes, and practices regarding ERAC. This cross-sectional study specifically aims to assess the current knowledge, attitudes, and practices regarding ERAC among obstetrics and gynecology medical staff in China.

## Materials and methods

### Study design and participants

This multi-center cross-sectional study was conducted from February 22 to March 31, 2025, across 10 hospitals located in eastern, central, and southern regions of China, coordinated by author’s Hospital. The study population comprised healthcare professionals working in departments of obstetrics, gynecology, and anesthesiology. Ethical approval was obtained from the Ethics Committee of author’s Hospital, and all participants provided informed consent prior to data collection.

Eligible participants included medical staff from the aforementioned departments who were fully informed about the study objectives and voluntarily agreed to participate. Individuals were excluded if they were interns or temporary staff with less than 3 months of clinical experience, or if they were on leave or had resigned during the study period.

### Questionnaire

The development of the questionnaire was informed by relevant literature (14) and established clinical guidelines, including the *Guidelines for postoperative care in cesarean delivery: Enhanced Recovery After Surgery (ERAS) Society recommendations (part 3)* (15) and the *Society for Obstetric Anesthesia and Perinatology: Consensus Statement and Recommendations for Enhanced Recovery After Cesarean* (8). Following the completion of the initial draft, feedback was sought from domain experts in obstetrics and anesthesiology to enhance the content validity of the questionnaire. Two senior anesthesiologists and two senior obstetricians, each with more than 10 years of clinical experience, were invited to review the instrument. Based on their feedback, several rounds of revisions were undertaken to refine the study design and participant inclusion criteria, standardize the wording of questionnaire items, and improve the content of the second section to ensure that the questions were applicable across different departments and professional roles. A pilot study involving 71 participants was conducted to evaluate the psychometric properties of the instrument. Reliability analysis indicated excellent internal consistency, with an overall Cronbach’s *α* coefficient of 0.946. The reliability of each dimension remained high, with Cronbach’s α values of 0.925, 0.941, and 0.964 for the knowledge, attitude, and practice domains, respectively. The Spearman–Brown split-half reliability coefficients were 0.757, 0.916, and 0.928 for the three domains, respectively. Construct validity was further examined using the Kaiser–Meyer–Olkin (KMO) test and confirmatory factor analysis (CFA). The KMO value was 0.960 (*p* < 0.001), indicating excellent sampling adequacy for factor analysis. CFA demonstrated an acceptable model fit (Supplementary Tables S1, S2), supporting the structural validity of the questionnaire. In addition, face validity was assessed during the pilot survey through participants’ feedback regarding the clarity and comprehensibility of the questionnaire items. Participants were invited to report any confusing or difficult items, and no significant issues were identified. Most participants indicated that the questionnaire was clear and easy to understand.

The finalized version of the questionnaire, administered in Chinese, comprised four major components: demographic characteristics, knowledge, attitudes, and practices dimensions (Supplementary Material Questionnaire). Demographic variables included age, gender, educational attainment, marital status, department affiliation, job title, length of service, professional rank, hospital type, hospital grade, and involvement in teaching or research activities, as well as prior participation in ERAC-related training. The knowledge section consisted of 10 conceptual items and 5 single-choice questions. For conceptual items, participants were asked to rate their familiarity with ERAC-related content using a three-point scale: “very familiar” (2 points), “heard of it” (1 point), and “unclear” (0 points). Each single-choice question had one correct answer, valued at 1 point. The total knowledge score ranged from 0 to 25. The attitude dimension included 10 statements evaluated on a five-point Likert scale, ranging from “strongly agree” to “strongly disagree,” with scores assigned from 5 to 1. Thus, the total possible score for the attitude section ranged from 10 to 50. The practice dimension comprised 10 behavior-related items, also scored on a five-point Likert scale, with response options from “never” (1 point) to “always” (5 points), resulting in the same scoring range of 10 to 50. Based on conventional Bloom’s threshold in KAP research (16, 17), cut-off scores for defining good knowledge, positive attitudes, and proactive practices were established at 80% or higher of the total possible score in each respective dimension.

### Questionnaire distribution and quality control

The study employed a convenience sampling strategy and utilized an online survey format for data collection. The questionnaire was developed through Wenjuanxing, a widely used Chinese online survey platform^1^, which generated a corresponding QR code for participant access. Each participating hospital designated a research coordinator responsible for managing the distribution process within their institution. Dissemination of the questionnaire was carried out through a combination of departmental WeChat groups and in-person distribution during regular departmental meetings to ensure broad outreach among eligible staff. To maintain data quality, strict exclusion criteria were applied. Questionnaires were deemed invalid and excluded from the analysis if the recorded completion time was less than 120 s, if the embedded attention-check question (Item: “Please select ‘b. Have heard of it’ for this item”) was answered incorrectly, or if response patterns exhibited mechanical repetition indicative of inattentive or automated answering behavior.

### Statistical analysis

Data analysis was performed using SPSS version 26.0 (IBM Corp., Armonk, NY, United States) and AMOS version 24.0 (IBM Corp., Armonk, NY, United States). For continuous variables, data were expressed as mean ± standard deviation (SD). Normality of the score distributions for each dimension was assessed using the Shapiro–Wilk test. For two-group comparisons, the independent *t*-test or Wilcoxon–Mann–Whitney test was used based on data normality. For comparisons among three or more groups, one-way ANOVA or the Kruskal–Wallis test was applied according to the assumptions of normality and homogeneity of variance. Spearman rank correlation analyses were conducted to examine the associations among the three core domains. Univariate and multivariate logistic regression analyses were conducted to identify the independent influence factors of knowledge, attitude, and practice scores. Variables with a *p*-value less than 0.05 in univariate logistic analysis were subsequently included in the multivariate models. Guided by the theoretical KAP framework, structural equation modeling (SEM) was employed to test the hypothesized mediating role of attitude in the relationship between knowledge and practice. The SEM analysis examined the hypotheses that (H1) knowledge is associated with attitude, (H2) knowledge is associated with practice, and (H3) knowledge is indirectly associated with practice through attitude. Both direct and indirect effects were estimated and compared to evaluate the strength and nature of the mediation pathways. A two-sided *p*-value < 0.05 was considered indicative of statistical significance throughout all analyses.

## Results

### Demographic information on participants

Initially, a total of 891 questionnaires were collected. The following were excluded: (1) Six participants who did not provide informed consent; (2) Three questionnaires with illogical age responses; (3) Seventy-three responses with incorrect answers to the trap question; (4) Twenty responses from non-gynecology/obstetrics medical technicians or graduate students; (5) Twenty-three questionnaires with a completion time of less than 120 s. After exclusions, 766 valid questionnaires remained, with an effective rate of 85.97%. Among them, 455 (59.40%) were physicians, 685 (89.43%) were female, 351 (45.82%) were aged 31–40 years, 524 (68.41%) had a bachelor’s degree, 176 (22.98%) had ≥21 years of work experience, and 276 (36.03%) had attended ERAC training. Participants’ demographic characteristics and KAP scores are summarized in Table 1.

**Table 1:** Basic characteristics and KAP scores of participants.

Characteristic	*N* (%)	Knowledge, mean ± SD	*P*	Attitude, mean ± SD	*P*	Practice, mean ± SD	*P*
*N* = 766							
Total score		14.64 ± 5.93		44.18 ± 5.45		32.33 ± 10.30	
Gender			**0.002**		0.909		0.777
Male	81(10.57%)	16.38 ± 6.11		44.41 ± 5.59		32.91 ± 9.46	
Female	685(89.43%)	14.43 ± 5.88		44.15 ± 5.44		32.26 ± 9.46	
Age			**0.006**		**0.013**		0.054
18–30 years	149(19.45%)	13.32 ± 5.53		43.23 ± 5.54		32.17 ± 10.63	
31–40 years	351(45.82%)	14.42 ± 6.00		44.04 ± 5.60		31.80 ± 10.07	
41–50 years	200(26.11%)	15.47 ± 5.93		44.61 ± 5.20		32.38 ± 10.60	
>50 years	66(8.62%)	16.32 ± 5.75		45.71 ± 4.80		35.41 ± 9.43	
Education			**0.003**		0.581		0.782
Associate degree	31 (4.05%)	13.42 ± 4.70		43.97 ± 4.91		32.77 ± 11.31	
Bachelor’s degree	524 (68.41%)	14.29 ± 5.74		44.10 ± 5.51		32.33 ± 10.36	
Master’s degree	154(20.10%)	15.49 ± 6.54		44.67 ± 5.36		31.94 ± 9.79	
Ph. D.	57(7.44%)	16.23 ± 6.05		43.67 ± 5.45		33.14 ± 10.74	
Marital status			**0.01**		**0.017**		0.246
Married	604(78.85%)	14.99 ± 5.97		44.40 ± 5.47		32.53 ± 10.39	
Other	162(21.15%)	13.35 ± 5.60		43.36 ± 5.30		31.59 ± 9.94	
Department			**0.005**		0.087		**<0.001**
Obstetrics	425(55.48%)	14.94 ± 5.94		44.37 ± 5.45		33.84 ± 9.82	
Gynecology	156(20.37%)	13.82 ± 5.72		43.96 ± 5.87		29.87 ± 11.81	
Obstetrics and Gynecology Rotation	83(10.84%)	13.18 ± 5.72		42.95 ± 5.07		30.81 ± 9.94	
Anesthesiology	102(13.32%)	15.84 ± 6.04		44.73 ± 4.99		31.06 ± 9.01	
Position			**0.034**		0.451		0.304
Physician	455(59.40%)	15.10 ± 6.07		44.36 ± 5.31		32.75 ± 9.98	
Nurse	232(30.29%)	13.99 ± 5.80		44.04 ± 5.68		31.98 ± 11.15	
Midwife	79(10.31%)	13.87 ± 5.24		43.52 ± 5.60		30.99 ± 9.41	
Years of work experience			**0.001**		**0.002**		0.139
≤5 years	119(15.54%)	13.18 ± 5.44		42.98 ± 5.20		31.13 ± 10.54	
6 ~ 10 years	186(24.28%)	13.78 ± 5.95		43.52 ± 5.64		31.83 ± 9.87	
11–20 years	285(37.21%)	15.04 ± 5.81		44.48 ± 5.66		32.51 ± 10.28	
≥21 years	176(22.98%)	15.89 ± 6.13		45.20 ± 4.84		33.39 ± 10.56	
Professional title			**<0.001**		**0.002**		**0.006**
Junior title or below	255(33.29%)	13.64 ± 5.78		43.40 ± 5.53		31.59 ± 10.98	
Intermediate title	312(40.73%)	14.51 ± 5.76		44.11 ± 5.59		31.70 ± 9.54	
Senior title (including associate senior and full senior titles)	199(25.98%)	16.14 ± 6.11		45.28 ± 4.95		34.28 ± 10.33	
Nature of hospital			0.119		0.917		0.059
Public hospital	742(96.87%)	14.58 ± 5.93		44.18 ± 5.44		32.22 ± 10.30	
Private hospital	24(3.13%)	16.46 ± 5.79		44.00 ± 5.44		35.75 ± 9.77	
Type of hospital			**0.002**		0.143		0.796
Specialized Obstetrics and Gynecology Hospital	559(72.98%)	14.95 ± 5.96		44.39 ± 5.50		32.26 ± 10.26	
General Hospital	207(27.02%)	13.80 ± 5.77		43.61 ± 5.30		32.53 ± 10.41	
Hospital level			**0.014**		0.146		0.581
Primary hospital	29(3.79%)	13.10 ± 5.14		43.17 ± 4.72		30.76 ± 12.79	
Secondary hospital	156(20.37%)	13.99 ± 5.16		43.49 ± 5.53		32.96 ± 9.67	
Tertiary hospital	581(75.85%)	14.89 ± 6.14		44.41 ± 5.45		32.24 ± 10.33	
Teaching responsibilities			**<0.001**		**0.002**		0.093
Yes	331(43.21%)	16.02 ± 5.94		44.86 ± 5.42		33.13 ± 9.94	
No	435(56.79%) (67.64%)	13.59 ± 5.71		43.66 ± 5.42		31.73 ± 10.53	
Research responsibilities			**<0.001**		**0.008**		**0.007**
Yes	236(30.81%)	16.42 ± 6.12		45.06 ± 5.15		33.87 ± 9.94	
No	530(69.19%)	13.85 ± 5.67		43.78 ± 5.54		31.65 ± 10.39	
ERAC training			**<0.001**		**<0.001**		**<0.001**
Yes	276(36.03%)	18.18 ± 5.07		45.65 ± 5.07		37.84 ± 8.80	
No	490(63.97%)	12.64 ± 5.43		43.35 ± 5.49		29.23 ± 9.78	

Bold values indicate statistical significance (*p* < 0.05).

### Knowledge, attitude, and practice

The distribution of knowledge dimensions showed that the three questions with the lowest number of participants choosing the ‘unfamiliar’ option were ‘ERAC is a postoperative enhanced recovery method following cesarean section (K1)’ with 17.36% and ‘ERAC helps mothers resume daily activities and improve quality of life by reducing analgesic use (K14)’ with 14.49%, and ‘The main components of ERAC include reduced fasting time, multimodal analgesia, infection prevention, early mobilization, and early feeding (K3)’ with 14.23%. For items involving the correctness rate, the items with the lowest correctness rates were item regarding the postoperative pain management (K6) with 26.50% (Supplementary Table S3). Responses to the attitude dimension showed that only 38.64% strongly agreed that implementing the ERAC pathway does not increase the additional workload (A10), only 40.08% strongly agreed that ERAC is a treatment approach suitable for all cesarean section patients (A8), and only 42.05% strongly agreed that ERAC is beneficial for neonatal safety (A4) (Supplementary Table S4). Responses to the practice dimension showed that 25.33% occasionally and 15.14% never participate in the development of ERAC-related clinical pathways or treatment plans (P5), 23.24% occasionally and 10.97% never have regular ERAC-related training or discussions in their department (P4), 23.24% occasionally and 10.05% never pay attention to the psychological status of patients after cesarean section and involve mental health professionals and family members in the care planning (P7) (Supplementary Table S5).

Additional subgroup analyses were conducted to explore potential differences in KAP item scores across departments, years of experience, and hospital types. The detailed results are presented in Supplementary Table S6.

### Univariate and multivariate analysis

Using 80% of the total score as the cutoff, the number of participants classified as good knowledge, positive attitude, and good practice were 201, 625, and 228, respectively. Multivariate logistic regression analysis showed that being a physician (OR = 2.682, 95% CI: 1.238–5.811, *p* = 0.012), having research responsibilities (OR = 1.675, 95% CI: 1.057–2.656, *p* = 0.028), and receiving ERAC training (OR = 6.369, 95% CI: 4.323–9.382, *p* < 0.001) were independently associated with good knowledge (Table 2). Regarding attitude, only higher knowledge scores were independently associated with positive attitudes (OR = 1.103, 95% CI: 1.063–1.145, *p* < 0.001) (Table 3). For practice, both knowledge (OR = 1.155, 95% CI: 1.108–1.205, *p* < 0.001) and attitude (OR = 1.147, 95% CI: 1.100–1.197, *p* < 0.001) scores were positively associated proactive practice. In addition, education level, department, hospital type, and ERAC training are all independently associated with practice levels (all *p* < 0.001) (Table 4).

**Table 2:** Univariate and multivariate analysis for knowledge dimension.

Variable	Univariate logistic regression		Multivariate logistic regression	
	OR (95%CI)	*P*	OR (95%CI)	*P*
Gender				
Male	1.878(1.162–3.037)	0.010	1.432(0.775–2.647)	0.353
Female	ref		ref	
Age				
18–30	0.340(0.178–0.650)	0.001	0.815(0.223–2.985)	0.758
31–40	0.515(0.297–0.892)	0.018	0.586(0.211–1.624)	0.304
41–50	0.659(0.370–1.176)	0.159	0.581(0.275–1.227)	0.155
>50	ref		ref	
Education				
Associate degree	ref		ref	
Bachelor’s degree	2.049(0.703–5.969)	0.189	1.465(0.456–4.702)	0.521
Master’s degree	3.542(1.177–10.656)	0.024	2.075(0.573–7.519)	0.267
Ph. D.	4.243(1.307–13.766)	0.016	2.767(0.678–11.290)	0.156
Marital status				
Married	1.826(1.177–2.832)	0.007	1.438(0.819–2.526)	0.206
Other	ref		ref	
Department				
Obstetrics	0.744(0.470–1.180)	0.209	1.151(0.625–2.120)	0.652
Gynecology	0.494(0.281–0.868)	0.014	0.780(0.389–1.562)	0.482
Obstetrics and Gynecology Rotation	0.422(0.211–0.844)	0.015	0.581(0.251–1.344)	0.204
Anesthesiology	ref		ref	
Position				
Physician	2.021(1.097–3.724)	0.024	2.682(1.238–5.811)	0.012
Nurse	1.243(0.644–2.400)	0.517	1.804(0.848–3.839)	0.126
Midwife	ref		ref	
Years of work experience				
≤5 years	ref		ref	
6 ~ 10 years	1.313(0.723–2.384)	0.370	1.082(0.480–2.436)	0.849
11–20 years	2.000(1.160–3.447)	0.013	1.481(0.527–4.158)	0.456
≥21 years	2.560(1.444–4.540)	0.001	1.536(0.438–5.380)	0.502
Professional title				
Junior title or below	ref		ref	
Intermediate title	1.462(0.976–2.191)	0.066	0.911(0.474–1.752)	0.780
Senior title (including associate senior and full senior titles)	2.553(1.668–3.908)	<0.001	0.829(0.335–2.050)	0.685
Nature of hospital				
Public hospital	0.582(0.251–1.351)	0.208		
Private hospital	ref			
Type of hospital				
Specialized Obstetrics and Gynecology Hospital	1.294(0.890–1.882)	0.177		
General Hospital	ref			
Hospital level				
Primary hospital	ref			
Secondary hospital	1.308(0.421–4.066)	0.642		
Tertiary hospital	2.585(0.886–7.540)	0.082		
Teaching responsibilities				
Yes	2.222(1.601–3.082)	<0.001	1.161(0.734–1.836)	0.524
No	ref		ref	
Research responsibilities				
Yes	2.541(1.816–3.556)	<0.001	1.675(1.057–2.656)	0.028
No	ref			
ERAC training				
Yes	6.151(4.332–8.734)	<0.001	6.369(4.323–9.382)	<0.001
No	ref		ref	

Knowledge scores were categorized using 80% of the total possible score as the cutoff; a score ≥20 indicated good knowledge (*n* = 201), while a score <20 indicated poor knowledge (*n* = 565).

**Table 3:** Univariate and multivariate analysis for attitude dimension.

Variable	Univariate logistic regression		Multivariate logistic regression	
	OR (95%CI)	*P*	OR (95%CI)	*P*
Knowledge	1.114(1.078–1.152)	<0.001	1.103(1.063–1.145)	<0.001
Gender				
Male	0.707(0.408–1.226)	0.217		
Female	ref			
Age				
18–30	0.372(0.156–0.888)	0.026	0.649(0.153–2.744)	0.557
31–40	0.503(0.220–1.150)	0.104	0.811(0.235–2.799)	0.740
41–50	0.647(0.270–1.547)	0.327	0.778(0.292–2.073)	0.616
>50	ref			
Education				
Associate degree	ref			
Bachelor’s degree	0.724(0.247–2.121)	0.556		
Master’s degree	0.565(0.184–1.731)	0.317		
Ph. D.	0.380(0.115–1.259)	0.113		
Marital status				
Married	1.358(0.887–2.079)	0.159		
Other	ref			
Department				
Obstetrics	0.964(0.541–1.720)	0.902		
Gynecology	0.806(0.420–1.549)	0.518		
Obstetrics and Gynecology Rotation	0.630(0.305–1.300)	0.211		
Anesthesiology	ref			
Position				
Physician	1.414(0.809–2.470)	0.224		
Nurse	2.044(1.092–3.826)	0.025		
Midwife	ref			
Years of work experience				
≤5 years	ref		ref	
6 ~ 10 years	1.055(0.611–1.820)	0.848	0.905(0.449–1.827)	0.781
11–20 years	1.558(0.920–2.638)	0.099	1.100(0.465–2.602)	0.828
≥21 years	1.949(1.065–3.565)	0.030	1.044(0.372–3.334)	0.942
Professional title				
Junior title or below	ref			
Intermediate title	1.158(0.770–1.741)	0.481	0.900(0.515–1.572)	0.711
Senior title (including associate senior and full senior titles)	2.052(1.220–3.450)	0.007	1.292(0.566–2.946)	0.543
Nature of hospital				
Public hospital	1.868(0.760–4.595)	0.173		
Private hospital	ref			
Type of hospital				
Specialized Obstetrics and Gynecology Hospital	0.995(0.659–1.503)	0.983		
General Hospital	ref			
Hospital level				
Primary hospital	ref			
Secondary hospital	0.807(0.286–2.282)	0.686		
Tertiary hospital	0.956(0.356–2.563)	0.928		
Teaching responsibilities				
Yes	1.330(0.914–1.936)	0.136		
No	ref			
Research responsibilities				
Yes	1.257(0.835–1.891)	0.273		
No	ref			
ERAC training				
Yes	2.082(1.366–3.174)	<0.001	1.189(0.738–1.915)	0.476
No	ref		ref	

Attitude scores were classified using 80% of the total score as the cutoff; a score ≥40 indicated a positive attitude (*n* = 625), while a score <40 indicated a negative attitude (*n* = 141).

**Table 4:** Univariate and multivariate analysis for practice dimension.

Variable	Univariate logistic regression		Multivariate logistic regression	
	OR (95%CI)	*P*	OR (95%CI)	*P*
Knowledge	1.219(1.177–1.261)	<0.001	1.155(1.108–1.205)	<0.001
Attitude	1.202(1.158–1.248)	<0.001	1.147(1.100–1.197)	<0.001
Gender				
Male	1.204(0.737–1.966)	0.458		
Female				
Age				
18–30	0.710(0.386–1.303)	0.269		
31–40	0.626(0.361–1.085)	0.095		
41–50	0.720(0.402–1.287)	0.267		
>50	ref			
Education				
Associate degree	ref		ref	
Bachelor’s degree	0.519(0.250–1.080)	0.079	0.292(0.123–0.696)	0.005
Master’s degree	0.426(0.193–0.942)	0.035	0.171(0.063–0.461)	<0.001
Ph. D.	0.516(0.208–1.278)	0.153	0.230(0.071–0.752)	0.015
Marital status				
Married	1.323(0.893–1.962)	0.163		
Other	ref			
Department				
Obstetrics	1.903(1.140–3.177)	0.014	2.557(1.348–4.850)	0.004
Gynecology	1.340(0.743–2.416)	0.331	2.080(1.010–4.282)	0.047
Obstetrics and Gynecology Rotation	1.007(0.498–2.035)	0.985	1.995(0.852–4.672)	0.112
Anesthesiology	ref			
Position				
Physician	1.271(0.737–2.192)	0.389		
Nurse	1.301(0.729–2.321)	0.373		
Midwife	ref			
Years of work experience				
≤5 years	ref			
6 ~ 10 years	0.987(0.584–1.669)	0.962		
11–20 years	1.310(0.811–2.116)	0.270		
≥21 years	1.431(0.855–2.396)	0.172		
Professional title				
Junior title or below	ref			
Intermediate title	0.889(0.614–1.287)	0.533		
Senior title (including associate senior and full senior titles)	1.444(0.972–2.146)	0.069		
Nature of hospital				
Public hospital	0.411(0.182–0.928)	0.032	0.243(0.087–0.682)	0.007
Private hospital	ref		ref	
Type of hospital				
Specialized Obstetrics and Gynecology Hospital	0.820(0.582–1.155)	0.256		
General Hospital	ref			
Hospital level				
Primary hospital	ref			
Secondary hospital	1.862(0.714–4.857)	0.204		
Tertiary hospital	1.599(0.640–3.996)	0.315		
Teaching responsibilities				
Yes	1.149(0.841–1.569)	0.382		
No	ref			
Research responsibilities				
Yes	1.440(1.037–2.000)	0.029	1.209(0.767–1.906)	0.413
No	ref		ref	
ERAC training				
Yes	4.703(3.381–6.541)	<0.001	2.312(1.551–3.446)	<0.001
No	ref		ref	

Practice scores were classified using 80% of the total score as the cutoff; a score ≥40 indicated good practice (*n* = 228), while a score <40 indicated poor practice (*n* = 538).

### Correlation analysis

Correlation analysis indicated significant positive correlations between knowledge and attitude (*r* = 0.501, *p* < 0.001), as well as practice (*r* = 0.471, *p* < 0.001). Meanwhile, there was also a significant correlation between attitude and practice (*r* = 0.441, *p* < 0.001) (Supplementary Table S7).

### SEM analysis

**Figure 1:**
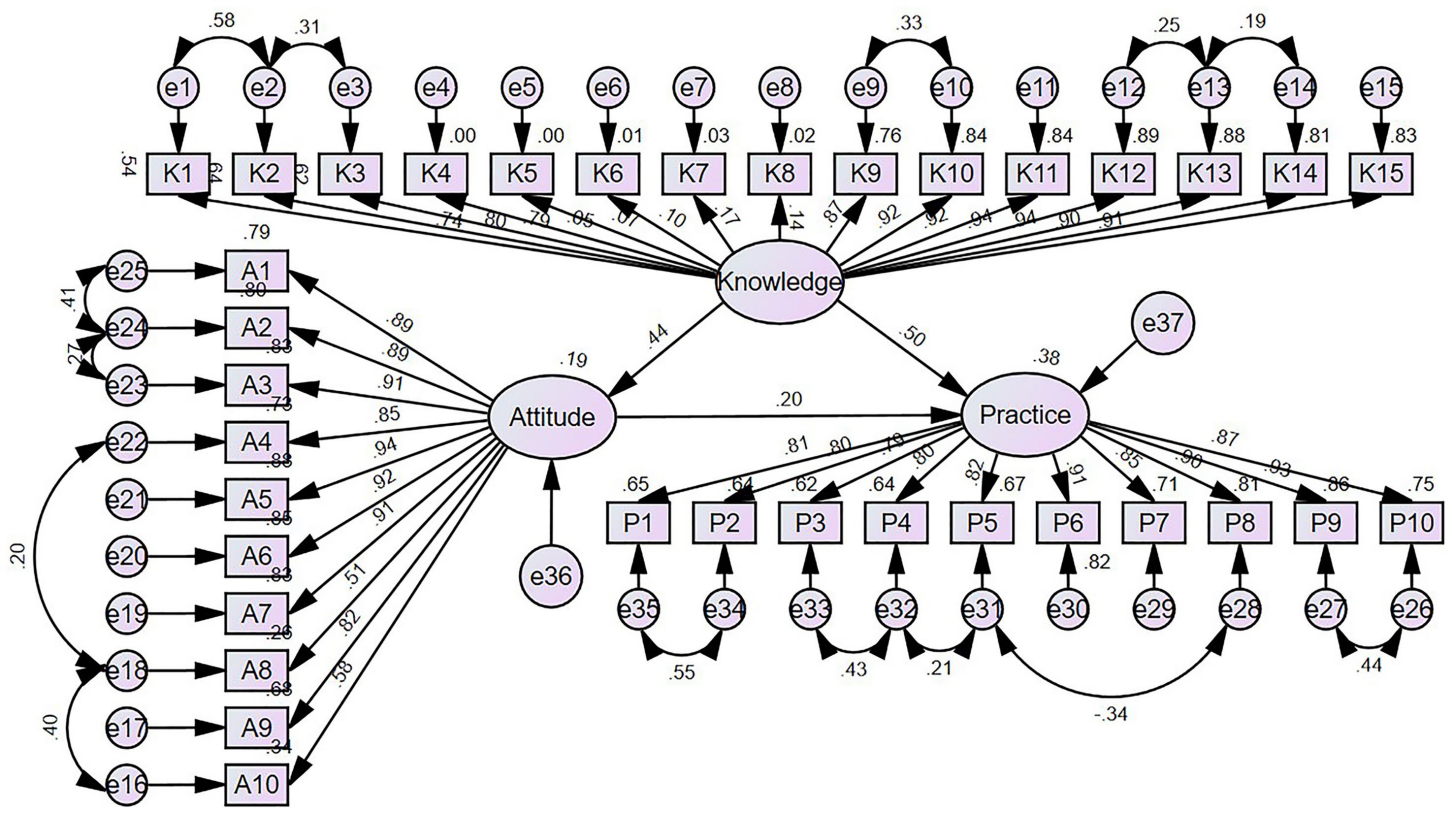
Structural equation model (SEM) depicting the relationships among the latent variables: knowledge, attitude, and practice. Ellipses represent latent variables (unobserved constructs), including overall knowledge, attitude, and practice. Rectangles represent observed variables (measured questionnaire items) that serve as indicators of the latent variables. Circles represent error terms or residuals associated with the observed variables. Single-headed arrows indicate hypothesized directional relationships (pathways) between latent variables and from latent variables to observed variables, with standardized path coefficients labeled on the arrows.

The fit of the SEM analysis showed good model fit indices (Supplementary Table S8). The SEM results suggested that knowledge was significantly associated with attitude (*β* = 0.441, *p* = 0.009) and practice (β = 0.501, *p* = 0.005), while attitude was also associated with practice (*β* = 0.203, *p* = 0.008). In addition, knowledge showed an indirect association with practice through attitude (*β* = 0.089, *p* = 0.006) (Supplementary Table S9 and Figure 1).

## Discussion

Despite generally positive attitudes, obstetrics and gynecology medical staff demonstrated insufficient knowledge and suboptimal implementation of ERAC, with significant disparities influenced by training, academic background, and institutional factors. To optimize ERAC implementation, targeted professional development initiatives—particularly standardized ERAC training—should be prioritized to bridge knowledge gaps and promote evidence-based practice across diverse clinical settings.

This study revealed a complex yet coherent pattern across the domains of KAP regarding ERAC among obstetrics and gynecology medical staff. Survey-based studies among healthcare providers in maternal care settings—particularly in China—have shown that although ERAC is conceptually acknowledged, actual implementation varies substantially, with less than 30% of providers consistently applying core ERAC components such as early mobilization and multimodal analgesia (18, 19). Previous research has indicated that healthcare professionals often endorse the value of ERAS programs but face multiple barriers when translating knowledge into sustained behavior change, particularly in obstetric contexts where traditions and risk perceptions remain entrenched. Previous studies have shown that although many obstetric professionals support ERAC principles, the actual implementation of key components, such as multidisciplinary collaboration and early mobilization, remains limited, often due to institutional barriers, resource constraints, and concerns regarding clinical risk (12).

A central theme emerging from the present findings is the insufficiency of ERAC-related knowledge among frontline staff in obstetrics and gynecology. While most respondents had at least heard of ERAC, only a minority demonstrated a clear understanding of its core components or multidisciplinary nature. This mirrors observations from similar studies in other departments, where superficial awareness did not equate to operational knowledge (20, 21). Notably, staff with prior ERAC training and academic or research involvement were more likely to exhibit stronger knowledge scores, suggesting that institutional exposure and structured learning opportunities significantly enhance familiarity. However, this advantage was not uniformly distributed, with nurses, midwives, and those in rotational roles showing lower levels of understanding. The uneven distribution of knowledge across roles may reflect longstanding disparities in professional development access and departmental communication, particularly in large hospital systems where protocol dissemination often remains physician-centric (22, 23). Integrating ERAC modules into mandatory in-service training and linking participation to credential renewal, supported by peer-led workshops and structured mentoring, may promote sustained knowledge uptake beyond one-off seminars.

Despite these knowledge gaps, the study found that attitudes toward ERAC were generally positive. Most respondents expressed confidence in ERAC’s capacity to improve recovery, reduce hospital stay, and enhance maternal wellbeing. These findings are consistent with previous research in surgical and maternal health contexts, where healthcare providers generally support patient-centered recovery models, particularly when benefits to safety and efficiency are emphasized (24, 25). However, the results also uncovered areas of ambivalence. A significant proportion of respondents were uncertain about whether ERAC added to their workload or could be universally applied to all cesarean patients. This echo concerns raised in qualitative studies, where staff cited institutional inertia, lack of administrative support, and fear of clinical risk as deterrents to full-scale implementation (26, 27). Interestingly, in our study, prior ERAC training was the only variable independently associated with more favorable attitudes, suggesting that exposure to structured content may help reframe perceived barriers and clarify misconceptions. Frequent, context-specific dialogues and interdisciplinary platforms for reflection and adaptation may enhance morale and foster deeper acceptance of ERAC practices.

The most prominent gap was observed in the practice domain, where scores lagged behind both knowledge and attitude. The results reflected a substantial disconnect between support for ERAC and its application in daily routines. Only a small proportion of staff consistently reported implementing ERAC-related practices, participating in protocol development, or receiving regular training. These findings are in line with prior work showing that clinical endorsement does not automatically translate into behavioral compliance, particularly in complex care pathways requiring interdepartmental coordination (28, 29). Multivariate analysis suggested that institutional setting and role responsibilities influenced practice, with research-active staff and those in private institutions more likely to implement ERAC principles. Conversely, staff with higher academic degrees appeared less engaged in practice, possibly due to shifts toward non-clinical duties or administrative roles. These patterns reveal that structural support, including routine audits, feedback loops, and integration of ERAC into electronic records, is essential for translating protocols into consistent practice.

Although each KAP component has distinct determinants, these domains are interrelated. Structural equation modeling suggested that knowledge was associated with practice both directly and indirectly through attitudes. This layered relationship supports existing behavioral models, including the Knowledge-Attitude-Practice framework and the Theory of Planned Behavior, which emphasize that knowledge may provide the cognitive basis for belief systems, while attitudes may mediate motivation toward behavior (30). Our findings lend weight to this model, highlighting that knowledge alone is not sufficient; efforts must also target the underlying belief structures that drive or inhibit action. This is particularly relevant in systems where protocol changes require departmental buy-in, shared expectations, and logistical adjustments. Similar observations have been reported in ERAS studies across different surgical specialties, where effective interdisciplinary collaboration and continuous professional engagement are associated with higher protocol adherence and improved implementation outcomes (7).

Additional subgroup analyses revealed significant differences in several KAP items across departments, years of professional experience, and hospital types. In particular, multiple knowledge items (e.g., K1–K3, K6–K7, and K10–K12) varied significantly by department and clinical experience, suggesting that familiarity with ERAC concepts may depend on professional roles and accumulated clinical exposure. This finding is consistent with previous studies indicating that successful ERAS/ERAC implementation requires coordinated understanding among multidisciplinary teams and continuous professional education to ensure consistent perioperative practices (31, 32). Differences observed in several practice items across departments further suggest that the implementation of ERAC-related behaviors may be influenced by departmental workflows and team-based perioperative management strategies. Overall, these findings highlight that both professional background and institutional context may contribute to variability in ERAC knowledge and practice among healthcare providers.

To optimize ERAC adoption, reforms must occur at multiple levels. At the policy level, administrative leaders should integrate ERAC metrics into performance assessments and allocate resources to ensure functional infrastructure. At the organizational level, departments must prioritize ongoing education, designate implementation champions, and build multidisciplinary teams. Clinically, protocols should be embedded in patient pathways, and staff given real-time feedback on adherence and outcomes. In addition, future research should examine how social norms, departmental leadership styles, and peer influence affect ERAC uptake. Exploring qualitative perspectives alongside longitudinal tracking would provide a richer understanding of barriers and enablers, helping to inform more tailored interventions (33, 34).

This study has several limitations. First, as a cross-sectional survey, it captures participants’ responses at a single point in time, limiting the ability to infer causal relationships between knowledge, attitudes, and practices. Second, the use of self-reported questionnaires may introduce social desirability bias, potentially leading to overestimation of positive attitudes or reported practices. In addition, knowledge, attitudes, and practices were measured using the same instrument, which may introduce common-method bias and potentially inflate the observed correlations among these variables. Third, although the sample included hospitals from multiple regions, the findings may not be generalizable to all obstetrics and gynecology staff across China due to potential regional or institutional differences in ERAC implementation and training opportunities. Fourth, because the survey was distributed through an online electronic questionnaire platform, the exact number of individuals who received the survey invitation could not be determined. Consequently, the response rate could not be accurately calculated, which may limit the ability to assess the representativeness of the sample.

In conclusion, although obstetrics and gynecology medical staff demonstrated generally positive attitudes toward ERAC, their limited knowledge and suboptimal implementation of relevant practices indicate substantial gaps in translating awareness into clinical behavior. To improve ERAC implementation, targeted training programs and continuous professional development initiatives may help enhance both knowledge and practice.

## Data Availability

The original contributions presented in the study are included in the article/Supplementary material, further inquiries can be directed to the corresponding authors.

## Footnotes

1
https://www.wjx.cn/
