# Supplementary material for: ERAC knowledge, attitudes, and practices among obstetrics and gynecology medical staff
Source: Front Public Health. 2026 Apr 10;14:1786598. doi: 10.3389/fpubh.2026.1786598 (PMC13106389; doi:10.3389/fpubh.2026.1786598)
Supplement: Supplementary file 1 [file Table_1.DOCX]

| Questionnaire ID: |
| --- |
| Dear Participant,  We are researchers from the Obstetrics and Gynecology Hospital of Fudan University, and we sincerely invite you to participate in our research project. This study aims to understand the **knowledge, attitudes, and practices** **of obstetric and gynecologic healthcare professionals regarding Enhanced Recovery After Cesarean Section (ERAC)**, in order to provide evidence for developing scientific intervention strategies. This research may help more people in the future and improve their health outcomes. Your participation in this study is voluntary, and the study has been reviewed and approved by the Ethics Committee. If you agree to participate, please read the following instructions carefully.  1. Please complete the questionnaire. There are no right or wrong answers — simply respond according to your actual situation. If you encounter any questions during completion, feel free to contact us. After finishing, please submit the questionnaire promptly.  2. This study involves a simple questionnaire survey and will not cause any physical or psychological harm. However, it includes some personal information such as your gender and age. We will strictly protect your privacy and ensure that your information will not be disclosed. Please fill it out with confidence.  3. As a participant, you have the right to be informed about the research and its progress at any time. If you decide to withdraw from the study, please notify us, and your data will not be included in the final analysis.  Finally, we sincerely thank you for taking the time out of your busy schedule to support our scientific research!  □I have read and understood the information above and agree that the collected data may be used for scientific research.  Signature of Informed Consent:  Date of Participation: ____ Year ____ Month ____ Day |

| **Part 1 Basic Information** | | | |
| --- | --- | --- | --- |
| 1. **Gender:** | a. Male  b. Female | | |
| 1. **Age: ____ years old** | | |  |
| 1. **Educational level:** | a. Associate degree  b. Bachelor’s degree  c. Master’s degree  d. Doctoral degree | | |
| 1. **Marital status:** | a. Unmarried  b. Married  c. Divorced  d. Widowed | | |
| 1. **Department:** | a. Obstetrics  b. Gynecology  c. Rotating between Obstetrics and Gynecology  d. Anesthesiology  e. Other (please specify) | | |
| 1. **Job position:** | a. Physician  b. Nurse  c. Midwife  d. Other (please specify) | | |
| 1. **Work experience:** | a. ≤ 5 years  b. 6–10 years  c. 11–20 years  d. ≥ 21 years | | |
| 1. **Professional title:** | a. Junior level or below  b. Intermediate level  c. Senior level (including associate senior and full senior titles) | | |
| 1. **Type of hospital you work in:** | a. Public hospital  b. Private hospital | | |
| 1. **Type of institution:** | a. Specialized obstetrics and gynecology hospital  b. General hospital | | |
| 1. **Hospital grade:** | a. Primary hospital  b. Secondary hospital  c. Tertiary hospital | | |
| 1. **Do you have teaching responsibilities?** | | a. Yes  b. No | |
| 1. **Do you have research responsibilities?** | | a. Yes  b. No | |
| 1. **Have you attended any training related to Enhanced Recovery After Cesarean Section (ERAC) in the past three years (including online or offline sessions)?** | | a. Yes  b. No | |

| **Part 2 Knowledge of Enhanced Recovery After Cesarean Section (ERAC)**  Please evaluate the following items based on your personal experience and level of understanding: a. Very familiar (have conducted in-depth study or research); b. Have heard of it (but have not studied or learned it in depth); c. Not familiar (have not paid attention to this topic) | | | |  |
| --- | --- | --- | --- | --- |
| **For the following statements, please indicate your level of understanding:** | | | | |
| 1. **ERAC is a treatment approach for enhanced recovery after cesarean section that promotes postoperative recovery of the mother through multiple measures.** | a. very familiar | b. have heard of it | c. not familiar | |
| 1. **The core goal of ERAC is to optimize maternal and neonatal clinical outcomes through a standardized, evidence-based, multidisciplinary collaborative process, thereby reducing postoperative stress and complications, shortening hospital stay, and improving patient satisfaction.** | a. very familiar | b. have heard of it | c. not familiar | |
| 1. **The main rehabilitation measures included in ERAC are reduced fasting time, multimodal analgesia, infection prevention, early mobilization, and early oral intake.** | a. very familiar | b. have heard of it | c. not familiar | |
| 1. **How long should fasting and fluid restriction be before surgery?**   **A. Fasting for 8 hours before surgery, no restriction on water B. Fasting for 6 hours before surgery, no fluids for 2 hours C. No water intake and fasting for 12 hours before surgery D. No fasting or fluid restriction before surgery E. None of the above is correct** |  |  |  | |
| 1. **What should preoperative health education for parturients include?**   **A. Explaining the cesarean section procedure and potential risks to the patient B. Informing the patient about postoperative pain management and ERAC goals C. Informing the patient of postoperative activity limitations and recovery time before surgery D. All of the above are correct E. None of the above are correct** |  |  |  | |
| 1. **Regarding postoperative pain management, which of the following is correct?**   **A. Multimodal analgesia should be adopted B. Only one of NSAIDs or acetaminophen should be used C. NSAIDs should be used only as needed; if the patient feels no pain, they are unnecessary D. All of the above are correct E. None of the above are correct** |  |  |  | |
| 1. **How soon can the patient begin eating after surgery?**   **A. Oral intake of liquids is allowed 1 hour after surgery**  **B. May eat 6 hours after surgery**  **C. May eat 12 hours after surgery D. May eat 48 hours after surgery E. None of the above are correct** |  |  |  | |
| 1. **What is the recommended practice for early postoperative mobilization?**   **A. The patient should get out of bed and move as soon as possible to avoid prolonged bed rest B. The patient must lie flat without a pillow for 8 hours after surgery and remain on bed rest C. No activity is allowed within 24 hours after surgery; complete bed rest is required D. Light activity may begin 48 hours after surgery E. None of the above are correct** |  |  |  | |
| 1. **ERAC can effectively reduce postoperative complications such as infection and thrombosis after cesarean section.** | a. very familiar | b. have heard of it | c. not familiar | |
| 1. **The implementation steps of ERAC include preoperative preparation, postoperative pain control, and early mobilization.** | a. very familiar | b. have heard of it | c. not familiar | |
| 1. **ERAC helps improve maternal mental health and alleviates postpartum anxiety and depression.** | a. very familiar | b. have heard of it | c. not familiar | |
| 1. **ERAC can significantly enhance postpartum recovery speed, particularly in pain management.** | a. very familiar | b. have heard of it | c. not familiar | |
| 1. **Compared with traditional postoperative recovery methods, ERAC can improve maternal satisfaction.** | a. very familiar | b. have heard of it | c. not familiar | |
| 1. **ERAC helps patients resume daily activities and improve quality of life by reducing the use of analgesic drugs.** | a. very familiar | b. have heard of it | c. not familiar | |
| 1. **ERAC requires multidisciplinary collaboration, including effective cooperation among teams from obstetrics, anesthesiology, maternal-fetal medicine, neonatology, nursing, nutrition, pharmacy, and hospital administration.** | a. very familiar | b. have heard of it | c. not familiar | |
| 1. **Please select “b. Have heard of it” for this item.** | a. very familiar | b. have heard of it | c. not familiar | |

Note: The answers to each question are marked in red font.

| **Part 3 Attitudes Toward Enhanced Recovery After Cesarean Section (ERAC)** | | | | | |
| --- | --- | --- | --- | --- | --- |
| 1. **I believe that ERAC can improve the recovery speed of patients after cesarean section.** | a. strongly agree | b. agree | c. neutral | d. disagree | e. strongly disagree |
| 1. **I believe that ERAC can help shorten the length of hospital stay for postpartum patients.** | a. strongly agree | b. agree | c. neutral | d. disagree | e. strongly disagree |
| 1. **I believe that ERAC helps reduce the incidence of postoperative complications.** | a. strongly agree | b. agree | c. neutral | d. disagree | e. strongly disagree |
| 1. **I believe that ERAC is beneficial for neonatal safety.** | a. strongly agree | b. agree | c. neutral | d. disagree | e. strongly disagree |
| 1. **I believe that ERAC can improve maternal mental health and reduce the risk of postpartum depression.** | a. strongly agree | b. agree | c. neutral | d. disagree | e. strongly disagree |
| 1. **I believe that ERAC pathways can improve overall patient satisfaction.** | a. strongly agree | b. agree | c. neutral | d. disagree | e. strongly disagree |
| 1. **I believe that the implementation of ERAC can improve hospital obstetric management efficiency.** | a. strongly agree | b. agree | c. neutral | d. disagree | e. strongly disagree |
| 1. **I believe that ERAC is a treatment approach suitable for all cesarean section patients.** | a. strongly agree | b. agree | c. neutral | d. disagree | e. strongly disagree |
| 1. **I believe that sufficient training and support are required for the implementation of ERAC.** | a. strongly agree | b. agree | c. neutral | d. disagree | e. strongly disagree |
| 1. **I believe that implementing ERAC pathways does not increase the additional workload.** | a. strongly agree | b. agree | c. neutral | d. disagree | e. strongly disagree |

| **Part 4 Practices Related to Enhanced Recovery After Cesarean Section (ERAC)**  Always: can be achieved almost 100% of the time; Often: can be achieved approximately ≥70% of the time; Sometimes: can be achieved approximately 40–70% of the time; Rarely: can be achieved approximately 10–40% of the time; Never: can be achieved less than 10% of the time or almost never | | | | | |
| --- | --- | --- | --- | --- | --- |
| 1. **In clinical practice, I often introduce ERAC-related information to postpartum patients.** | a. always | b. often | c. sometimes | d. rarely | e. never |
| 1. **I provide postpartum recovery care in accordance with ERAC-related guidelines.** | a. always | b. often | c. sometimes | d. rarely | e. never |
| 1. **In my actual work, I have participated in the implementation and operation of ERAC.** | a. always | b. often | c. sometimes | d. rarely | e. never |
| 1. **In my department, ERAC-related training or discussions are held regularly.** | a. always | b. often | c. sometimes | d. rarely | e. never |
| 1. **I participate in the development of clinical pathways or treatment plans related to ERAC.** | a. always | b. often | c. sometimes | d. rarely | e. never |
| 1. **I adjust the implementation of ERAC according to the patient’s specific condition.** | a. always | b. often | c. sometimes | d. rarely | e. never |
| 1. **I pay attention to the psychological state of postpartum patients and invite psychologists and family members to participate in the care plan.** | a. always | b. often | c. sometimes | d. rarely | e. never |
| 1. **When implementing ERAC, I take into account the patient’s individual needs and preferences.** | a. always | b. often | c. sometimes | d. rarely | e. never |
| 1. **I regularly evaluate the effectiveness of ERAC implementation and make corresponding improvements.** | a. always | b. often | c. sometimes | d. rarely | e. never |
| 1. **When the outcomes of ERAC are unsatisfactory, I take the initiative to identify possible causes and communicate with other team members.** | a. always | b. often | c. sometimes | d. rarely | e. never |

| **Thank you once again for participating in our survey. The information you provided is invaluable to our future work!**  **Thank you for filling out our questionnaire！**  Should you have any feedback or suggestions regarding this research, we would be honored to hear from you.  Feedback/Suggestions： （optional）  To ensure this survey effectively contributes to advancing our follow-up efforts, we would greatly appreciate it if you could provide your contact information.  Your Phone Number: （optional） |
| --- |
