# Supplementary material for: ERAC knowledge, attitudes, and practices among obstetrics and gynecology medical staff
Source: Front Public Health. 2026 Apr 10;14:1786598. doi: 10.3389/fpubh.2026.1786598 (PMC13106389; doi:10.3389/fpubh.2026.1786598)
Supplement: Supplementary file 2 [file Table_2.DOCX]

**Supplementary table 1. Fitting Results of CFA Model**

| Model | Ref. | Measured results |
| --- | --- | --- |
| CMIN/DF | 1-3 excellent，3-5 good | 4.822 |
| IFI | >0.8 good | 0.926 |
| TLI | >0.8 good | 0.919 |
| CFI | >0.8 good | 0.925 |

**Supplementary table 2. The effect values of each path in CFA**

|  |  |  | Estimate | S.E. | C.R. | P |
| --- | --- | --- | --- | --- | --- | --- |
| K1 | <--- | Knowledge | 1 |  |  |  |
| K2 | <--- | Knowledge | 1.066 | 0.047 | 22.45 | <0.001 |
| K3 | <--- | Knowledge | 1.091 | 0.048 | 22.56 | <0.001 |
| K4 | <--- | Knowledge | 0.044 | 0.03 | 1.462 | 0.144 |
| K5 | <--- | Knowledge | 0.057 | 0.03 | 1.879 | 0.06 |
| K6 | <--- | Knowledge | 0.097 | 0.035 | 2.747 | 0.006 |
| K7 | <--- | Knowledge | 0.181 | 0.038 | 4.818 | <0.001 |
| K8 | <--- | Knowledge | 0.108 | 0.029 | 3.79 | <0.001 |
| K9 | <--- | Knowledge | 1.201 | 0.047 | 25.622 | <0.001 |
| K10 | <--- | Knowledge | 1.244 | 0.046 | 27.064 | <0.001 |
| K11 | <--- | Knowledge | 1.253 | 0.046 | 26.958 | <0.001 |
| K12 | <--- | Knowledge | 1.321 | 0.047 | 28.093 | <0.001 |
| K13 | <--- | Knowledge | 1.327 | 0.047 | 27.952 | <0.001 |
| A10 | <--- | Attitude | 1 |  |  |  |
| A9 | <--- | Attitude | 0.87 | 0.05 | 17.399 | <0.001 |
| A8 | <--- | Attitude | 0.987 | 0.06 | 16.329 | <0.001 |
| A7 | <--- | Attitude | 1.039 | 0.056 | 18.484 | <0.001 |
| A6 | <--- | Attitude | 1.03 | 0.056 | 18.389 | <0.001 |
| A5 | <--- | Attitude | 1.07 | 0.058 | 18.589 | <0.001 |
| A4 | <--- | Attitude | 1.039 | 0.058 | 17.847 | <0.001 |
| A3 | <--- | Attitude | 1.035 | 0.056 | 18.601 | <0.001 |
| A2 | <--- | Attitude | 1.015 | 0.055 | 18.397 | <0.001 |
| A1 | <--- | Attitude | 0.97 | 0.053 | 18.225 | <0.001 |
| P1 | <--- | Practice | 1 |  |  |  |
| P2 | <--- | Practice | 0.971 | 0.034 | 28.343 | <0.001 |
| P3 | <--- | Practice | 1.014 | 0.037 | 27.126 | <0.001 |
| P4 | <--- | Practice | 0.975 | 0.035 | 27.841 | <0.001 |
| P5 | <--- | Practice | 1.018 | 0.038 | 27.131 | <0.001 |
| P6 | <--- | Practice | 1.121 | 0.034 | 33.086 | <0.001 |
| P7 | <--- | Practice | 1.041 | 0.036 | 29.178 | <0.001 |
| P8 | <--- | Practice | 1.052 | 0.033 | 31.998 | <0.001 |
| P9 | <--- | Practice | 1.106 | 0.032 | 34.215 | <0.001 |
| P10 | <--- | Practice | 1.02 | 0.034 | 30.375 | <0.001 |
| K14 | <--- | Knowledge | 1.275 | 0.048 | 26.642 | <0.001 |
| K15 | <--- | Knowledge | 1.221 | 0.046 | 26.687 | <0.001 |

**Supplementary table 3. Distribution of knowledge dimension responses**

| Knowledge items. n (%) | Very familiar | Heard of it | Not sure |
| --- | --- | --- | --- |
| 1. ERAC is a postoperative enhanced recovery method following cesarean section that promotes maternal recovery through multiple approaches. | 162 (21.15%) | 471 (61.49%) | 133 (17.36%) |
| 2. The core objective of ERAC is to optimize clinical outcomes for both mothers and newborns by implementing a standardized, evidence-based, and multidisciplinary collaborative process, thereby reducing postoperative stress and complications, shortening hospital stays, and improving patient satisfaction. | 174 (22.72%) | 464 (60.57%) | 128 (16.71%) |
| 3. The main components of ERAC include reduced fasting time, multimodal analgesia, infection prevention, early mobilization, and early feeding. | 217 (28.33%) | 440 (57.44%) | 109 (14.23%) |
| 9. ERAC can effectively reduce the incidence of complications such as infection and thrombosis after cesarean section. | 249 (32.51%) | 431 (56.27%) | 86 (11.23%) |
| 10. The implementation steps of ERAC include preoperative preparation, postoperative pain control, and early mobilization. | 246 (32.11%) | 438 (57.18%) | 82 (10.70%) |
| 11. ERAC helps improve maternal mental health and alleviates postpartum anxiety and depression. | 277 (29.63%) | 444 (57.96%) | 95 (12.40%) |
| 12. The implementation of ERAC can significantly accelerate maternal postpartum recovery, especially in terms of pain management. | 237 (30.94%) | 431 (56.27%) | 98 (12.79%) |
| 13. Compared to traditional postoperative recovery approaches, ERAC improves maternal satisfaction. | 237 (30.94%) | 427 (55.74%) | 102 (13.32%) |
| 14. By reducing the use of analgesic medications, ERAC helps mothers resume daily activities and improve quality of life more quickly. | 221 (28.85%) | 434 (56.66%) | 111 (14.49%) |
| 15. ERAC requires multidisciplinary collaboration, and effective cooperation among obstetrics, anesthesia, maternal-fetal medicine, neonatology, nursing, nutrition, pharmacy, and hospital administration teams is essential. | 235 (30.68%) | 447 (58.36%) | 84 (10.97%) |
|  | Correctness |  |  |
| 4. Recommended duration for preoperative fasting and fluid restriction | 632 (82.51%) |  |  |
| 5. Key components of preoperative health education for pregnant women | 635 (82.90%) |  |  |
| 6. Knowledge of correct postoperative pain management strategies | 203 (26.50%) |  |  |
| 7. Timing for resuming oral intake after surgery | 256 (33.42%) |  |  |
| 8. Recommended practices for early postoperative mobilization | 651 (84.99%) |  |  |

**Supplementary table 4. Distribution of attitude dimension responses**

| Attitude items. n (%) | Strongly agree | Agree | Neutral | Disagree | Strongly disagree |
| --- | --- | --- | --- | --- | --- |
| 1. I believe that ERAC can enhance the recovery speed of patients after cesarean section. | 427 (55.74%) | 312 (40.73%) | 27 (3.25%) | 0 | 0 |
| 2. I believe that ERAC can help reduce the length of hospital stay for mothers. | 422 (55.09%) | 310 (40.47%) | 34 (4.44%) | 0 | 0 |
| 3. I believe that ERAC helps reduce the incidence of postpartum complications. | 419 (54.70%) | 331 (43.21%) | 55 (7.18%) | 1 (0.13%) |  |
| 4. I believe that ERAC is beneficial for neonatal safety. | 379 (42.05%) | 147 (26.87%) | 142 (25.96%) | 28 (5.12%) | 0 |
| 5. I believe that ERAC can improve maternal mental health and reduce the risk of postpartum depression. | 420 (54.83%) | 305 (39.82%) | 40 (5.22%) | 1 (0.13%) | 0 |
| 6. I believe that the ERAC pathway can improve overall patient satisfaction. | 427 (55.74%) | 303 (39.56%) | 35 (4.57%) | 1 (0.13%) | 0 |
| 7. I believe that the implementation of ERAC can improve the efficiency of obstetric management in hospitals. | 425 (55.48%) | 303 (39.56%) | 38 (4.96%) | 0 | 0 |
| 8. I believe that ERAC is a treatment approach suitable for all cesarean section patients. | 307 (40.08%) | 286 (37.34%) | 90 (11.75%) | 76 (9.92%) | 7 (0.91%) |
| 9. I believe that the implementation of ERAC requires adequate training and support. | 448 (58.49%) | 297 (38.77%) | 21 (2.74%) | 0 | 0 |
| 10. I believe that implementing the ERAC pathway does not increase the additional workload. | 296 (38.64%) | 303 (39.56%) | 120 (15.67%) | 44 (5.74%) | 3 (0.39%) |

**Supplementary table 5. Distribution of practice dimension responses**

| Practice items. n (%) | Always | Often | Sometimes | Occasionally | Never |
| --- | --- | --- | --- | --- | --- |
| 1. In clinical practice, I often introduce ERAC-related information to patients undergoing cesarean section. | 135 (17.62%) | 243 (31.72%) | 195 (25.46%) | 138 (18.02%) | 55 (7.18%) |
| 2. I provide postpartum recovery care in accordance with ERAC-related guidelines. | 159 (20.76%) | 262 (34.20%) | 191 (24.93%) | 106 (13.84%) | 48 (6.27%) |
| 3. In my actual work, I have participated in the implementation and operation of ERAC. | 129 (16.84%) | 201 (26.24%) | 193 (25.20%) | 167 (21.80%) | 76 (9.92%) |
| 4. In your department, are there regular ERAC-related trainings or discussions? | 86 (11.23%) | 181 (23.63%) | 237 (30.94%) | 178 (23.24%) | 84 (10.97%) |
| 5. I participate in the development of ERAC-related clinical pathways or treatment plans. | 91 (11.88%) | 159 (20.76%) | 206 (26.89%) | 194 (25.33%) | 116 (15.14%) |
| 6. I adjust the implementation of ERAC according to the specific conditions of each patient. | 124 (16.19%) | 213 (27.81%) | 203 (26.50%) | 153 (19.97%) | 73 (9.53%) |
| 7. I pay attention to the psychological status of patients after cesarean section and involve mental health professionals and family members in the care planning. | 112 (14.62%) | 193 (25.20%) | 206 (26.89%) | 178 (23.24%) | 77 (10.05%) |
| 8. When implementing ERAC, I take into account the individual needs and preferences of patients. | 155 (20.23%) | 272 (35.51%) | 183 (23.89%) | 103 (13.45%) | 53 (6.92%) |
| 9. I regularly evaluate the effectiveness of ERAC implementation and make corresponding improvements. | 125 (16.32%) | 232 (30.29%) | 209 (27.28%) | 139 (18.15%) | 61 (7.96%) |
| 10. When the effect of ERAC is unsatisfactory, I will take the initiative to explore the reasons and communicate with other team members for improvement. | 125 (16.32%) | 236 (30.81%) | 218 (28.46%) | 129 (16.84%) | 58 (7.57%) |

**Supplementary table 6. Subgroup comparisons of KAP questionnaire item scores by department, years of experience, and hospital type**

| Domain | Item | P value for department | P value for experience | P value for hospital type |
| --- | --- | --- | --- | --- |
| Knowledge | K1 | 0.012 | <0.001 | 0.62 |
|  | K2 | 0.005 | 0.012 | 0.361 |
|  | K3 | 0.003 | 0.001 | 0.639 |
|  | K4 | 0.41 | 0.39 | <0.001 |
|  | K5 | 0.144 | 0.158 | <0.001 |
|  | K6 | 0.003 | 0.328 | <0.001 |
|  | K7 | 0.001 | 0.086 | <0.001 |
|  | K8 | 0.541 | 0.606 | 0.372 |
|  | K9 | 0.124 | 0.005 | 0.469 |
|  | K10 | 0.003 | <0.001 | 0.731 |
|  | K11 | 0.136 | 0.002 | 0.136 |
|  | K12 | 0.021 | <0.001 | 0.676 |
|  | K13 | 0.157 | <0.001 | 0.843 |
|  | K14 | 0.033 | 0.009 | 0.93 |
|  | K15 | 0.096 | 0.011 | 0.62 |
| Attitude | A1 | 0.09 | 0.006 | 0.026 |
|  | A2 | 0.129 | 0.002 | 0.001 |
|  | A3 | 0.174 | <0.001 | 0.008 |
|  | A4 | 0.222 | 0.001 | 0.085 |
|  | A5 | 0.096 | <0.001 | 0.129 |
|  | A6 | 0.035 | 0.001 | 0.03 |
|  | A7 | 0.778 | 0.008 | 0.011 |
|  | A8 | 0.01 | 0.047 | 0.9 |
|  | A9 | 0.218 | 0.008 | 0.022 |
|  | A10 | 0.001 | 0.002 | 0.797 |
| Practice | P1 | 0.001 | 0.001 | 0.862 |
|  | P2 | <0.001 | 0.007 | 0.411 |
|  | P3 | 0.009 | 0.032 | 0.581 |
|  | P4 | 0.001 | 0.868 | 0.163 |
|  | P5 | <0.001 | 0.912 | 0.043 |
|  | P6 | 0.004 | 0.358 | 0.755 |
|  | P7 | <0.001 | 0.398 | 0.152 |
|  | P8 | 0.003 | 0.066 | 0.992 |
|  | P9 | <0.001 | 0.294 | 0.591 |
|  | P10 | 0.004 | 0.704 | 0.353 |

**Supplementary table 7. Correlation analysis**

|  | Knowledge | Attitude | Practice |
| --- | --- | --- | --- |
| Knowledge | 1 |  |  |
| Attitude | 0.501 (P<0.001) | 1 |  |
| Practice | 0.471 (P<0.001) | 0.441 (P<0.001) | 1 |

**Supplementary table 8. SEM fit indicators**

| Model 1 | Ref. | Measured results |
| --- | --- | --- |
| CMIN/DF | 1-3 excellent，3-5 good | 3.305 |
| IFI | >0.8 good | 0.956 |
| TLI | >0.8 good | 0.951 |
| CFI | >0.8 good | 0.956 |

**Supplementary table 9. Analysis of direct and indirect effects in SEM**

| Model paths | Standardized direct effects  (95%CI) | P | Standardized indirect effects  (95%CI) | P |
| --- | --- | --- | --- | --- |
| Knowledge → Attitude | 0.441 (0.386-0.497) | 0.009 |  |  |
| Knowledge → Practice | 0.501 (0.442-0.575) | 0.005 | 0.089 (0.060-0.127) | 0.006 |
| Attitude → Practice | 0.203 (0.134-0.272) | 0.008 |  |  |
